# Therapeutic targeting truncated adenomatous polyposis coli (APC) proteins for the selective killing of colorectal cancer cells

**DOI:** 10.1186/1471-2164-15-S2-O3

**Published:** 2014-04-02

**Authors:** Lu Zhang, Sang B Kim, Ugur Eskiocak, Bruce Posner, Priyabrata Das, Woodring E Wright, Jef K De Brabander, Jerry W Shay

**Affiliations:** 1 Department of Cell Biology and Biochemistry, The University of Texas Southwestern Medical Center, Dallas, TX 75390, USA; 2 Children's Research Institute, The University of Texas Southwestern Medical Center, Dallas, TX 75390, USA; 3 King Abdulaziz University, Centre of Excellence in Genomic Medicine Research, 80216 Jeddah 21589, Kingdom of Saudi Arabia

## 

The adenomatous polyposis coli (APC) gene is a multifunctional tumor suppressor mutated in over 80% of colorectal cancers (CRC). Mutations in APC are believed to be one of the earliest events that contribute to CRC initiation. APC has been implicated in the negative regulation of the canonical Wnt signaling pathway, cell cycle control, cell migration, differentiation, and apoptosis. However, there remain many unanswered questions about the precise roles of APC in CRC progression. For example, over 90% of APC mutations result in truncated proteins, among which termination mutations at codons 1309 and 1450 occur the most frequently. It is completely unclear why the vast majority of human colon cancers would retain truncated APC proteins unless there was some survival/growth advantage to the emerging tumor. We hypothesized that these truncated APC proteins may actually play a dominant (gain of function) role in the initiation and/or progression of CRC and have conducted experiments to test this idea. Our rationale for these experiments is there are no disease-specific targeted treatments for CRC and therapeutic agents targeting APC truncations could be beneficial.

We developed a unique series of telomerase (TERT) and CDK4 immortalized human colonic epithelial cell (HCEC) lines and experimentally made isogenic derivatives by ectopic expression of the most commonly observed APC truncations found in patients. In addition, we made TERT and CDK4 expressing HCECs with TP53 and APC knockdowns as well as ectopic expression of oncogenic KRASv12. We have completed a cell-based high-throughput screen within UT Southwestern's 200,000 compound library utilizing the most cancer progressed isogenic cell line and tested the initial hits against the TERT immortalized normal diploid HCECs. A small molecule lead compound, TASIN-1 (truncated APC selective inhibitor), was identified from this screen that is selectively toxic towards a panel of colon cancer cell lines with APC truncations while sparing normal diploid HCECs and cancer cells with wild type APC. TASIN-1 and its analogs (with IC50s from 0.01nM to 5µM) represent a novel strategy for the treatment of colon cancer. In vitro and in vivo preclinical results will be presented.

